# Long-segment spinal cord infarction complicated with multiple cerebral infarctions: a case report

**DOI:** 10.1186/s12883-022-02888-8

**Published:** 2022-09-22

**Authors:** Weifang Xing, Wensheng Zhang, Guozhong Ma, Guofen Ma, Jinzhao He

**Affiliations:** Department of Neurology, Heyuan People’s Hospital, Guangdong Provincial People’s Hospital Heyuan Hospital, Heyuan, 517000 Guangdong Province China

**Keywords:** Long-segment, Spinal cord infarction, Anterior spinal artery syndrome, Cerebral infarction, Magnetic resonance

## Abstract

**Background:**

Spinal cord infarction is a rare disorder, constituting only 1% to 2% of all neurological vascular emergencies (making it less frequent than ischaemic brain injury); however, it is severe. A case of long-segment spinal cord infarction complicated with multiple cerebral infarctions has not been reported to date.

**Case presentation:**

Here, we describe one such case: a patient with spinal cord infarction from the cervical 7 (C7) to thoracic 6 (T6) vertebrae, along with anterior spinal artery syndrome and complicated by multiple cerebral infarctions. A 65-year-old farmer experienced sudden onset of severe pain in his chest, back and upper limbs while unloading heavy objects. Subsequently, both his lower limbs became weak and hypoaesthetic, and he was unable to walk. Spinal magnetic resonance imaging (MRI) revealed equal T1 and long T2 signals centred on the anterior horn of the spinal cord. The axial slice of these signals was shaped like an owl’s eye. After receiving drug treatment and active rehabilitation treatment, the patient’s ability to walk was restored.

**Conclusions:**

Long-segment spinal cord infarction is rare and can be complicated with cerebral infarction. The specific aetiology is worth exploring.

## Background

The spinal cord is part of the central nervous system, and its associated vascular system can exhibit thrombosis, embolism, ischaemia, bleeding, congenital malformations, aneurysms and other pathologies. Spinal cord infarction is rare, accounting for approximately 0.3% to 1% of ischaemic strokes [1]. Long-segment spinal cord infarction is even more uncommon. This paper reports a rare case of long-segment spinal cord infarction complicated with multiple cerebral infarctions; this case exhibited unique imaging features, and the patient had a good prognosis after treatment.

## Case presentation

A 65-year-old farmer was admitted to Heyuan People’s Hospital on December 23, 2021, due to sudden weakness in both lower limbs lasting for 11 h. He had a history of hypertension that lasted for more than 20 years but did not regularly take his oral antihypertensive drugs. He had poor blood pressure control, with a blood pressure of 153/98 mmHg on admission. This patient had smoked an average of 1–2 packs/day for more than 40 years. Before admission, the patient had been carrying a heavy object on his shoulder and suddenly experienced severe pain in his chest, back and upper limbs at the moment when he put down the object. Subsequently, both his lower limbs became weak and hypoaesthetic, and he was unable to walk. However, his head, neck and upper limbs exhibited normal function and sensation. He suffered from sphincter disturbances, abdominal distension and obvious tenderness and percussion pain of the thoracic vertebrae. Superficial sensation was bilaterally lost below the level of the nipples, and the skin temperature of both lower limbs decreased. While the muscle strength of both upper limbs was grade 5, that of both lower limbs was grade 0. Similarly, the muscle tone of both upper limbs was normal, but that of both lower limbs decreased. Tendon reflexes of both lower limbs were lacking, and the patient’s bilateral pathological signs were negative.

**Fig. 1:**
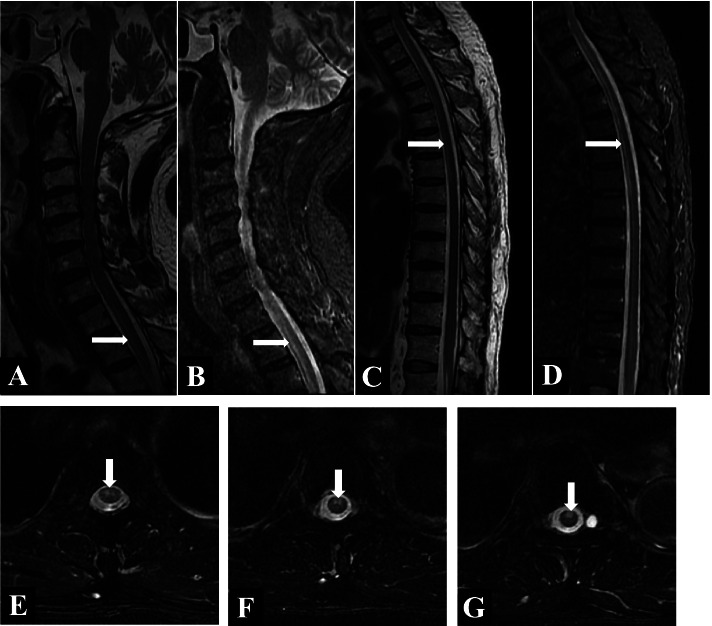
**A**-**G**: Symmetrical equal T1 and long T2 signal shadow can be seen in the cross section of the anterior horn of gray matter in the anterior part of the spinal cord at the level of C7–T6 vertebral body, showing “eagle eye” change. The sagittal position is parallel to the long axis of the spinal cord. No obvious enhancement lesion was found on enhanced scan. Spinal cord infarction at the level of C7–T6 vertebral body is considered

**Fig. 2:**
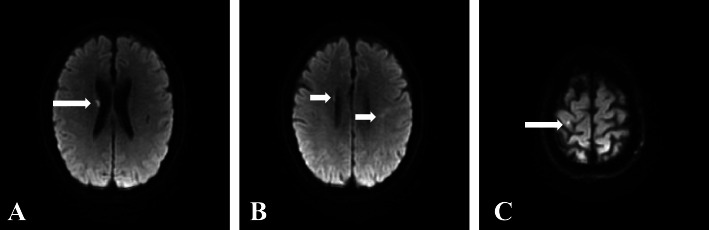
**A**-**C**: Acute lacunar infarction in the right basal segment, left corona radiata and right frontal lobe

**Fig. 3:**
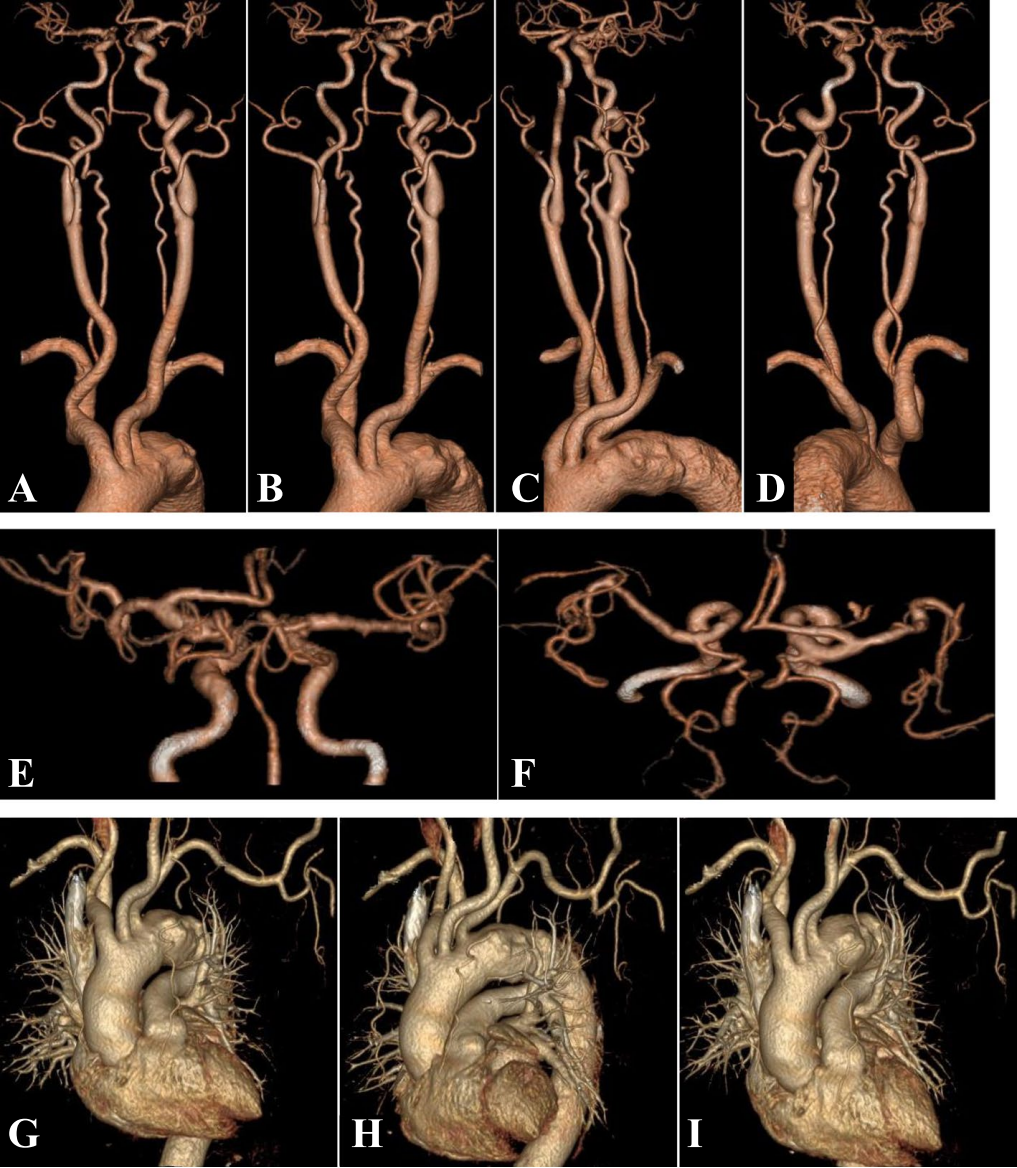
A-I: Head and neck CTA (computed tomography angiography), and chest CTA: noncalcified plaque of the aortic arch with mild stenosis and multiple penetrating ulcers, noncalcified plaque and mild stenosis of the descending aorta; Noncalcified plaque with mild luminal stenosis in the proximal segment of the left common carotid artery and the initial segment of the left internal carotid artery; mixed plaque in the left carotid sinus and mild luminal stenosis; Calcified plaque in the right carotid sinus and the C4–6 segment of the right internal carotid artery; and mild stenosis of the basilar artery

The patient underwent routine blood tests, including those for cytometry; liver and kidney function; blood glucose levels; glycosylated haemoglobin levels; blood lipid levels; coagulation function; pretransfusion parameters; C-reactive protein levels; procalcitonin levels; vasculitis indicators. He also underwent other laboratory tests or measurements. His results were all normal. Electrocardiogram (ECG) analysis showed a sinus rhythm and left ventricular high voltage. Cardiac colour Doppler ultrasound showed mild aortic and mitral regurgitation, normal left ventricular systolic function and decreased diastolic function. The M-type left ventricular ejection fraction was 64%. Enhanced MRI (magnetic resonance imaging) of the neck, chest and waist (Fig. 1 A–G) revealed the following: suspected spinal cord infarction from the cervical 7 (C7) to thoracic 6 (T6) vertebrae and disc herniation at C2/3, C3/4, C4/5 and C6/7 as well as at lumbar 4/5. Brain magnetic resonance imaging (MRI) (Fig. 2 A–C) revealed the following: acute lacunar cerebral infarction in the right basal segment, left corona radiata and right frontal lobe. Head and neck computed tomography angiography (CTA) and chest CTA (Fig. 3 A–I) revealed the following: noncalcified plaque of the aortic arch with mild stenosis and multiple penetrating ulcers, noncalcified plaque and mild stenosis of the descending aorta; noncalcified plaque with mild luminal stenosis in the proximal segment of the left common carotid artery and the initial segment of the left internal carotid artery; mixed plaque in the left carotid sinus and mild luminal stenosis; calcified plaque in the right carotid sinus and the C4–6 segment of the right internal carotid artery; and mild stenosis of the basilar artery.

After admission, we administered comprehensive treatment to the patient according to the principles of the treatment of cerebral infarction. Antiplatelet aggregation, lipid regulation and plaque stabilization therapy were the highest priorities. Drugs to enhance blood circulation and cerebroprotein hydrolysate were used to increase the blood supply to the ischaemic focus and nourish nerves. Additionally, glucocorticoid therapy was administered to reduce local inflammation and oedema. Mannitol was used to eliminate spinal cord oedema. We prescribed active rehabilitation treatment, such as acupuncture, electrical stimulation, barotherapy, and passive exercise, for this patient. Nursing care, such as regularly turning the patient over, monitoring indwelling urinary catheters, and assisting with defecation, was provided to prevent complications such as bedsores and urinary tract infections.

On December 31, 2021, the patient’s lower limb muscles regained the ability to contract, exhibiting involuntary twitches. The muscle strength of both lower limbs was scored as grade 1. On January 2, 2022, the patient regained the ability to move his lower limbs horizontally and experienced sensations of pain and temperature; the muscle strength of both lower limbs was scored as grade 2. On January 4, the patient reported the cessation of tenderness and percussion pain in the thoracic vertebra. Shallow bilateral hypoesthesia was present 2 cm under the level of the nipples. The muscle strength of both lower limbs remained at grade 2, and the muscle tone of both lower limbs was greater in the posterior portion than in the anterior portion. Both lower limbs exhibited a restored tendon reflex. By January 7, both lower limbs had regained some strength. The patient could move his lower limbs horizontally and flex them slightly. The muscle strength of both lower limbs reached grade 3. In a telephone follow-up on March 10, 2022, the patient reported that he could walk with the help of a four-legged walker, that he still experienced reduced sensations of pain and temperature in both lower limbs, and that he experienced abnormal sensations such as a continuous burning sensation. Additionally, the patient reported urinary dysfunction, managed by the use of a long-term indwelling catheter, and normal stool evacuation; together, these findings indicated that the prognosis of our patient was acceptable.

## Discussion and conclusions

The incidence of spinal vascular disease is much lower than that of cerebrovascular disease because the spinal cord is supplied with blood through several routes, with vascular redundancy. Specifically, the spinal cord is usually supplied with blood by 6 to 8 main root medullary arteries from the craniovertebral junction to the conus medullaris. This collateral circulation makes the spinal cord significantly more tolerant of ischaemia than brain tissue. However, the disadvantage of this redundant blood supply is the watershed region between the two arterial blood-supply areas. This watershed area has reduced blood supply and is thus more vulnerable to ischaemic damage. The blood flow in the cervical and lumbar segments of the spinal cord is significantly higher than that in the thoracic segment, especially the upper thoracic segment [2]. This paper reports a rare case of long-segment spinal cord infarction. The lesions involved the C6–T7 segment of the spinal cord, mainly covering the thoracic segment. The lesions were extensive and unusual.

The causes of spinal cord infarction include not only common factors, such as atherosclerosis, vasculitis, hypertension, dyslipidaemia, diabetes, hyperfibrinogen, and long-term heavy smoking, but also the following factors [3–6]: fibrocartilage embolism, congenital heart disease surgery or spinal cord decompression, aortic dissection aneurysm or aortic thrombosis, hypotension, percutaneous vertebroplasty, multiple rib fractures, spinal cord arteriovenous malformations, epidural abscess, epidural haematoma, intervertebral disc prolapse, adhesive arachnoiditis and bacterial meningitis.

In addition to the spinal cord MRI that suggested long-segment spinal cord infarction, the brain MRI also suggested multiple acute lacunar infarctions in this patient. Therefore, after further discussion, we determined that the most likely stroke mechanism was embolic infarction; such emboli originate from the atherosclerotic aortic arch. The following findings support this inference. First, the chest CTA showed that the aortic arch exhibited noncalcified plaque with stenosis and multiple ulcers; an external force could have knocked loose the noncalcified plaque when the patient set down the heavy object. Second, we did not find evidence of atrial fibrillation on the ambulatory ECG or obvious abnormality on the colour Doppler echocardiography, thus ruling out cardiogenic embolism. Third, the vascular anatomy could explain why the spinal cord infarction and cerebral infarction occurred simultaneously. The spinal cord infarction could have been caused by vertebral artery embolism, and the multiple cerebral infarctions could have been caused by internal carotid artery embolism. The multiple infarct lesions could also be explained by embolic infarction. However, this case suggests that there is merit to further exploration of the aetiology.

Spinal cord infarction is an ischaemic spinal vascular disease with a stroke-like onset; it often reaches a peak within a few minutes or hours. Given his clinical manifestations, this patient was considered to have anterior spinal artery syndrome, specifically, anterior 2/3 syndrome [4, 7], which is more common in the thoracic segment. Generally, the first symptom of this condition is severe nerve root pain located at the corresponding level of the lower boundary of the affected segment. Most cases involve dissociative sensory disorders with the loss of sensation involving pain and temperature and the retention of deep sensation. More obvious manifestations are dysfunctions of urination and defecation. After 3 months of follow-up, the neurological deficit in this patient was significantly improved. Therefore, we recommend that the initial treatment of such patients include medication and active rehabilitation treatment.

The incidence rate of spinal cord infarction is relatively low, and the aetiology and clinical manifestations of this disease are quite distinct. Clinically, spinal cord infarction is effectively differentiated from Guillain–Barre syndrome, acute myelitis, spinal cord demyelinating disease, spinal cord injury and other spinal cord diseases [8]. MRI examination is very important for the diagnosis of this disease and the assessment of the patient’s condition. Currently, there is no universal scheme for treating spinal cord infarction, and most therapies are based on the treatment scheme for cerebral infarction. With deepening clinical understanding of spinal cord infarction, the clinical treatment of this disease will improve.

In conclusion, long-segment spinal cord infarction is quite rare and can be complicated with cerebral infarction. The specific aetiology merits further exploration.

## Data Availability

Not applicable.

## Abbreviations

CTA: Computed tomography angiography
MRI: Magnetic resonance imaging
ECG: Electrocardiogram
